# Quantitative phantom‐based comparison of commercial CT metal artifact reduction algorithms

**DOI:** 10.1002/acm2.70631

**Published:** 2026-05-21

**Authors:** Beechui Koo, Daehong Kim, Hyunuk Jung, Mitchell Polizzi, Indra J. Das, Siyong Kim, James J. Sohn

**Affiliations:** ^1^ Department of Radiation and Cellular Oncology University of Chicago Chicago Illinois USA; ^2^ Data Science Institute University of Chicago Chicago Illinois USA; ^3^ Department of Radiological Science Kangwon National University Samcheok‐si Gangwon‐do South Korea; ^4^ Department of Radiation Oncology University of Rochester Rochester New York USA; ^5^ Department of Radiation and Oncology Virginia Commonwealth University Richmond Virginia USA; ^6^ Department of Radiation Oncology Northwestern Memorial Hospital, Northwestern University Feinberg School of Medicine Chicago Illinois USA

**Keywords:** CT, high‐z material, image quality, metal artifact reduction (MAR), metallic implants

## Abstract

**Background:**

Metal artifacts in computed tomography (CT) significantly compromise image quality and CT number (CTN) accuracy, which may influence downstream applications such as radiation therapy workflows. Although commercial metal artifact reduction (MAR) algorithms are widely used, their performance varies across vendors and clinical scenarios, and standardized methods for quantitative comparison remain limited.

**Purpose:**

This study quantitatively compares four commercial MAR algorithms using standardized metrics and a novel analytical approach.

**Methods:**

A custom‐designed resin phantom with interchangeable inserts (solid water, aluminum, titanium, and stainless steel) was scanned using four CT systems: Canon, GE, Philips, and Siemens. Each system's proprietary MAR algorithm was applied to obtain artifact‐reduced image sets. Quantitative analysis employed complementary methods: (1) circular profile analysis measuring mean CTN deviations at concentric distances from metal objects; (2) geometric accuracy assessment of metal objects; and (3) volumetric artifact characterization using analysis of severe artifact voxels and composite error metric (*M*‐error index) that integrates both artifact extent and intensity. Color maps and accumulated histograms of ΔCTN values between pre‐ and post‐MAR images were used to characterized artifact patterns and MAR performance.

**Results:**

All four MAR methods showed minimal |ΔCTN| at mid (2.80 cm) and far (3.80 cm) distances but showed pronounced increases in |ΔCTN| near the metal interface at the closest radius (1.27 cm). Smart‐MAR exhibited the highest CTN deviation for aluminum (16.83 HU), while O‐MAR showed the largest deviations for titanium and stainless steel (42.40 and 42.60 HU). Quantitative evaluation showed that single‐energy metal artifact reduction (SEMAR) and iterative metal artifact reduction (iMAR) achieved the most consistent geometric accuracy (<5%) across all metals. O‐MAR showed the highest accuracy for titanium (approximately 0.36%) but larger deviations for aluminum (approximately 9%), while Smart‐MAR exhibited the greatest errors exceeding 19% for both titanium and stainless steel. SEMAR, Smart‐MAR, and iMAR showed lower *M* Error values (0.00–0.83) than O‐MAR, which showed significantly higher values (1.15–2.45).

**Conclusion:**

SEMAR and iMAR achieved the most consistent geometric accuracy (<5% error) and artifact suppression across all metals, while O‐MAR demonstrated material‐dependent performance with substantial residual artifacts near metal interfaces. These phantom‐based benchmarks provide vendor‐neutral performance data on MAR performance under controlled settings.

## 1 INTRODUCTION

Metal artifacts represent one of the most significant challenges in computed tomography (CT) imaging for radiation oncology.^1^ These artifacts manifest as streaking, shadowing, and distortions radiating from metallic objects such as dental fillings, hip prostheses, and spinal implants, severely degrading image quality and potentially affecting image interpretation and introducing uncertainty in downstream applications such as target delineation and dose calculations in radiotherapy treatment planning. Previous studies have reported that metal artifacts can introduce substantial uncertainties in CT number (CTN) accuracy, which may affect downstream radiotherapy workflows. Uncorrected treatment plans can exhibit dose calculation errors exceeding 30% of the prescribed dose in regions affected by metal artifacts.^2^, ^3^

In response to this challenge, major CT manufacturers have developed proprietary metal artifact reduction (MAR) algorithms: single‐energy MAR (SEMAR) by Canon Medical Systems (Otawara, Japan),^4^ Smart‐MAR by GE Healthcare (Chicago, IL),^5^ Iterative MAR (iMAR) by Siemens Healthineers (Erlangen, Germany),^6^ and MAR for orthopedic implants (O‐MAR) by Philips Healthcare (Amsterdam, Netherlands).^7^ Generally, these algorithms operate by identifying and segmenting metal artifacts in the image or projection domain and applying corrections through iterative reconstruction, interpolation, or sinogram inpainting techniques. While these commercial solutions promise improved image quality, their implementation details remain undisclosed due to intellectual property considerations, creating a “black box” for end‐users who must rely on vendor claims rather than objective performance data.^8^

Previous comparative studies using commercial phantoms with multiple fixed inserts (e.g., Chou et al.) faced a fundamental limitation: when metal artifacts from multiple objects overlap, it becomes impossible to isolate whether observed performance differences reflect algorithm‐specific characteristics or simply the geometric configuration of overlapping artifact patterns. Our single‐insert design addresses this methodological challenge by enabling controlled, reproducible assessment of each algorithm's intrinsic performance characteristics without confounding multi‐scatter effects or artifact‐artifact interactions.^9^ Chou et al. compared four commercial MAR techniques using three types of metal implants but employed an acrylic phantom with fixed metal implants that limited the isolation of individual metal effects. Similarly, Hilgers et al. evaluated only Philips’ O‐MAR algorithm for hip prostheses without comparison to other vendors’ solutions.^7^ More critically, most comparative analyses have employed linear profile measurements, which capture artifacts only along specific directions and fail to characterize the complex, radially distributed nature of metal artifacts that typically propagate in all directions from metallic objects.^10^

The volumetric impact of these artifacts further complicates assessment, as metal‐induced distortions extend across multiple slices and affect three‐dimensional dose calculations in treatment planning.^11^, ^12^ Traditional evaluation methods that focus on single‐slice analysis or subjective visual assessment cannot fully capture these complexities, particularly when comparing algorithms that may exhibit different artifact patterns and reduction characteristics.

To address these limitations, we present a novel quantitative comparison of four commercial MAR algorithms using a custom‐designed phantom with interchangeable standardized inserts of varying densities. Our methodology introduces several key innovations: (1) circular profile analysis that measures artifact propagation at concentric distances around metal objects, providing a comprehensive radial assessment rather than directionally biased linear measurements; (2) volumetric artifact characterization that accounts for the three‐dimensional nature of metal artifacts across multiple slices; and (3) statistical distribution analysis through accumulated histograms that quantifies algorithm performance in restoring CTNs in artifact‐affected regions toward baseline values. This comprehensive assessment provides transparent, vendor‐neutral performance data on commercial MAR algorithms and establishes a controlled, quantitative benchmarking framework for evaluating MAR algorithm behavior under standardized conditions.

## 2 METHODS AND MATERIALS

### 2.1 Phantom design and materials

**FIGURE 1:**
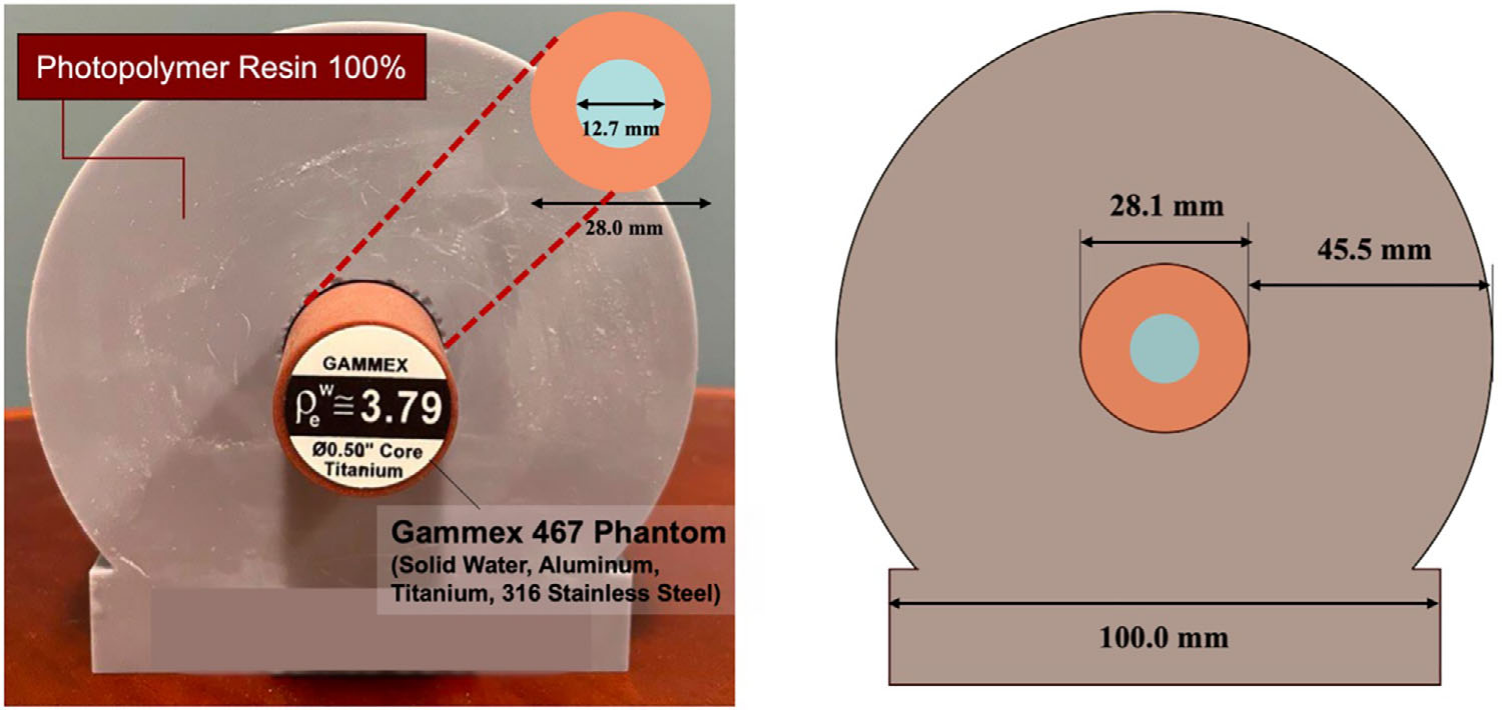
Custom cylindrical metal artifact phantom used in this study. (Left) Photograph of the phantom, consisting of a 3D‐printed photopolymer resin body (100% infill density) with a centrally embedded cylindrical metal insert. (Right) Schematic cross‐section of the 3D‐printed phantom design with the central metal insert channel. The total depth (axial length) of the phantom is 5 cm.

A customized in‐house cylindrical metal artifact phantom was fabricated using a Vat Photopolymerization (SLA/DLP) 3D printing technique with a commercial photopolymer resin (average density: 1.09 g/cm^3^; Anycubic Photon Mono X, Shenzhen, China) and 100% infill to ensure a solid, homogeneous structure. Although the resin is not equivalent to commercial solid water (∼1.04–1.06 g/cm^3^), its density approximates soft tissue and was considered suitable for relative CTN comparison and artifact evaluation.^13^ The phantom measures 11.9 cm in diameter and 5.0 cm in depth and contains a central cylindrical channel (28.0 mm diameter) for interchangeable rod inserts. Each insert is composed of a high‐density metal core (12.7 mm diameter) that is concentrically embedded within a solid water housing. Four standardized rods from the Gammex 467 Tissue Characterization Phantom (Gammex, Middleton, WI) were used in this study: (1) solid water (baseline control), (2) aluminum, (3) titanium, and (4) 316 stainless steel. These materials span a wide range of physical and electron densities relative to water, representing various metallic implants encountered in clinical practice. The rods were exchanged manually and scanned individually within the same phantom body to maintain consistent geometry across acquisitions. Figure 1 illustrates the phantom and rod insert design.

### 2.2 CT scanners and acquisition parameters

Four commercial CT scanners and their proprietary MAR algorithms were evaluated: Canon Aquilion Exceed LB with SEMAR, GE Discovery RT with Smart MAR, Philips Brilliance Big Bore with O‐MAR, and Siemens SOMATOM go.Open Pro with iMAR. For simplicity, these techniques are referred to as SEMAR, Smart‐MAR, O‐MAR, and iMAR, respectively, throughout this study.

Scans were acquired using standardized protocols where possible, including 120 kVp and matched tube current settings to achieve comparable dose levels (∼10 mGy CTDI_vol_ measured with a 32 cm body phantom). For each scanner–insert combination, images were reconstructed with and without MAR for paired comparison. Acquisition and reconstruction parameters, including field of view, rotation time, slice thickness, and soft tissue reconstruction kernels, are summarized in Table 1. Complete harmonization across vendors was not possible due to hardware and software constraints; slice thickness ranged from 1.00 mm (Siemens) to 3.00 mm (Philips), and field of view ranged from 130 × 130 mm (Canon) to 300 × 300 mm (Philips). Therefore, this study evaluates the integrated clinical performance of each vendor's MAR implementation as deployed in routine practice, rather than isolating algorithm‐specific effects from acquisition parameter influences.

**TABLE 1:** CT scan protocols for all phantom scans.

Parameter	Canon Medical Systems (Otawara, Japan)	GE Healthcare (Milwaukee, WI)	Siemens Healthcare (Erlangen, Germany)	Philips Healthcare (Best, Netherlands)
Scanner type	Canon Aquilion Exceed LB	GE Discovery RT	Siemens SOMATOM go.Open Pro	Philips Brilliance Big Bore
Slice thickness (mm)	2.00	1.25	3.00	1.00
Tube voltage (kVp)	120	120	120	120
Reconstruction kernel	FC41	Soft	B	Qr40f
Field of view	130×130	160×160	250×250	300×300
Rotation time (s)	0.5	0.5	0.5	0.5

### 2.3 Data analysis

Our analysis combined visual, radial profile‐based, and volumetric statistical methods to comprehensively characterize MAR algorithm performance.

#### 2.3.1 Color map visualization of CT number changes and pre‐MAR distributions

To visually assess the spatial distribution and severity of residual artifacts, difference maps were generated for each MAR technique by subtracting the MAR image from the corresponding non‐MAR image:

$$\Delta CTN\;=\mathrm{CT}N_{\mathrm{non}-\mathrm{MAR}}\;-\mathrm{CT}N_{\mathrm{MAR}}$$

To reduce the influence of isolated noise spikes and extreme outliers, the display range was clipped to the 3rd–97th percentile of ΔCTN values. A consistent color scale was applied across all scanner systems and MAR algorithms to enable direct visual comparison. Difference maps were generated separately for each insert material: solid water, aluminum, titanium, and stainless steel.

#### 2.3.2 CT number deviation analyses using circular profile

**FIGURE 2:**
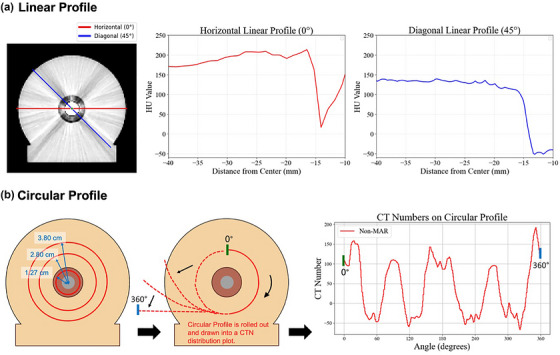
Comparison of traditional linear profile analysis and concentric circular profile analysis. (a) Linear profiles extracted along two directions (one along the 0° horizontal axis and another along a 45° diagonal axis) illustrating directional dependence when sampling CT numbers. (b) Concentric circular profile analysis used for metal artifact quantification. Red rings indicate the circular regions sampled for CT number (CTN) analysis at fixed radii from the center of the metal insert: 1.27, 2.80, and 3.80 cm. The circular profiles were “unwrapped” into a 2D representation to evaluate CT number variations. An example is shown for a stainless‐steel scan after applying Philips O‐MAR. CT, computed tomography; O‐MAR, metal artifact reduction for orthopedic implant.

To quantify metal artifacts in a rotationally symmetric manner, circular profiles were extracted concentrically around each metal insert. Unlike traditional line profiles, which can vary depending on line placement and may capture either dark or bright streaks, as shown in Figure 2A, circular profiles sample artifact effects across all directions. The circular profiles were measured at three fixed radial distances as illustrated in Figure 2B: 1.27 cm (near the metal), 2.80 cm (mid‐range), and 3.80 cm (far) from the insert center. These distances were selected to capture clinically relevant artifact behavior in radiation therapy planning: the immediate peri‐metal region where streak artifacts are most severe, the mid‐range region relevant to PTV extension, and the distal region where residual artifacts may affect organs‐at‐risk or background tissue dose calculations. Together, these distances span the typical range of artifact influence observed in clinical CT datasets (Huang et al.).^3^ For each scan, five consecutive axial slices centered on the metal insert were analyzed, and the mean ΔCTN across these slices was used to improve robustness against slice‐to‐slice variation. Mean absolute CTN deviations from the mean solid water baseline were calculated for each circular profile. This analysis was performed for both MAR‐corrected and non‐MAR reconstructions, enabling paired comparison of artifact reduction relative to a common reference baseline. Results are grouped by insert material type—aluminum, titanium, and stainless steel—to assess the dependence of MAR performance on metal attenuation.

#### 2.3.3 Metal geometric accuracy

The geometric accuracy of reconstructed metal objects was evaluated using a threshold‐based segmentation method, with the threshold set to 50% of the maximum CTN for each insert, adapted from Huang et al.^10^ For each scan, five consecutive slices centered near the insert mid‐plane were analyzed. Measured diameters were compared to the known physical dimension (12.7 mm), and percentage errors were calculated to quantify MAR‐related dimensional distortion. Mean values and standard deviations were reported to capture slice‐to‐slice variability.

#### 2.3.4 Volumetric analyses

Volumetric analysis was performed by applying a donut‐shaped mask to each axial slice of the CT scans for all MAR techniques. The number of analyzed slices varied by vendor due to varying slice thickness and image quality due to hardware limitations: Philips O‐MAR (6–8 slices), Canon SEMAR (10 slices), GE Smart‐MAR (24 slices), and Siemens iMAR (30 slices). The donut‐shaped mask used an inner radius of 15.0 mm and an outer radius of 45.0 mm, designed to exclude the central metal insert while remaining within the phantom boundary.

##### 2.3.4.1 Analysis of severe artifact voxels and composite error metric—M‐error index

The impact of artifacts was assessed volumetrically using annular cylindrical regions surrounding each metal insert. Voxels were classified as “bad” if their CTN deviated beyond a defined threshold of 40 HU from expected solid water values. The 40 HU threshold was selected based on prior dosimetric studies demonstrating that CTN deviations of this magnitude have been clinically relevant for treatment planning accuracy (Huang et al.).^3^ This error level approaches or exceeds the 3% dose calculation accuracy recommended by ICRU Report 24 and AAPM Task Group protocols for clinical treatment planning. Although the dosimetric impact may vary by tissue type and treatment site, the same threshold was applied consistently across all algorithms to enable standardized comparison. Severity of the artifact was quantified using the error metric *M*_error_ index, a composite metric combining both artifact extent and intensity, with lower values indicating superior MAR performance^10^:

$$\;M_{\mathrm{error}}=\left(\frac{\%\mathrm{bad}\;\mathrm{voxels}}{100}\right)\;\times \mathrm{mean}{\left|\Delta CTN\right|}_{\mathrm{bad}\;\mathrm{voxels}}$$

The percentage of bad voxels was calculated as the number of voxels exceeding the threshold divided by the total number of voxels within the annular region. This normalization reduces dependence on the absolute number of voxels analyzed, which varied across vendors due to differences in slice thickness. Multiplying this proportion by the mean absolute CTN deviation of bad voxels, $\mathrm{mean}{\left|\Delta CTN\right|}_{\mathrm{bad}\;\mathrm{voxels}}$, yields a single burden metric that increases with both the spatial extent and the magnitude of artifacts.

To quantify MAR‐related improvement, we additionally calculated Δ*M*_error_, the difference between the *M*_error_ values obtained from non‐MAR and MAR‐corrected images:

$$\Delta M_{\mathrm{error}}=M_{\mathrm{error}}\;\left(\mathrm{non}-\mathrm{MAR}\right)-M_{\mathrm{error}}\left(\mathrm{MAR}\right)$$

Higher Δ*M*_error_ values indicate greater improvement in artifact suppression relative to the non‐MAR baseline. Δ*M*_error_ enables a intuitive comparison of algorithm performance across different materials and scanner systems.

##### 2.3.4.2 Accumulated histogram of ΔCT number in annular region

To characterize the distribution of CTN deviations within the annular region, |ΔCTN| were binned using 5‐HU intervals and accumulated across all analyzed slices. The resulting accumulated histogram (cumulative distribution function) represents the fraction of voxels with ΔCTN values less than or equal to each threshold. Steeper curves indicate tighter clustering of CTN values and more consistent artifact suppression, whereas gradual curves indicate broader CTN deviation and greater artifact variability. The horizontal position of the curve relative to ΔCTN = 0 HU reflects systematic bias, with shifts toward negative or positive values indicating CTN underestimation or overestimation, respectively. All analyses were implemented using Python (NumPy, SciPy, OpenCV) and 3D Slicer software. Automated contouring algorithm was performed using cv2.findContours in OpenCV to improve consistency across evaluations, with manual refinement applied when residual artifacts or segmentation ambiguity affected accuracy.

## 3 RESULTS

### 3.1 Color map visualization

**FIGURE 3:**
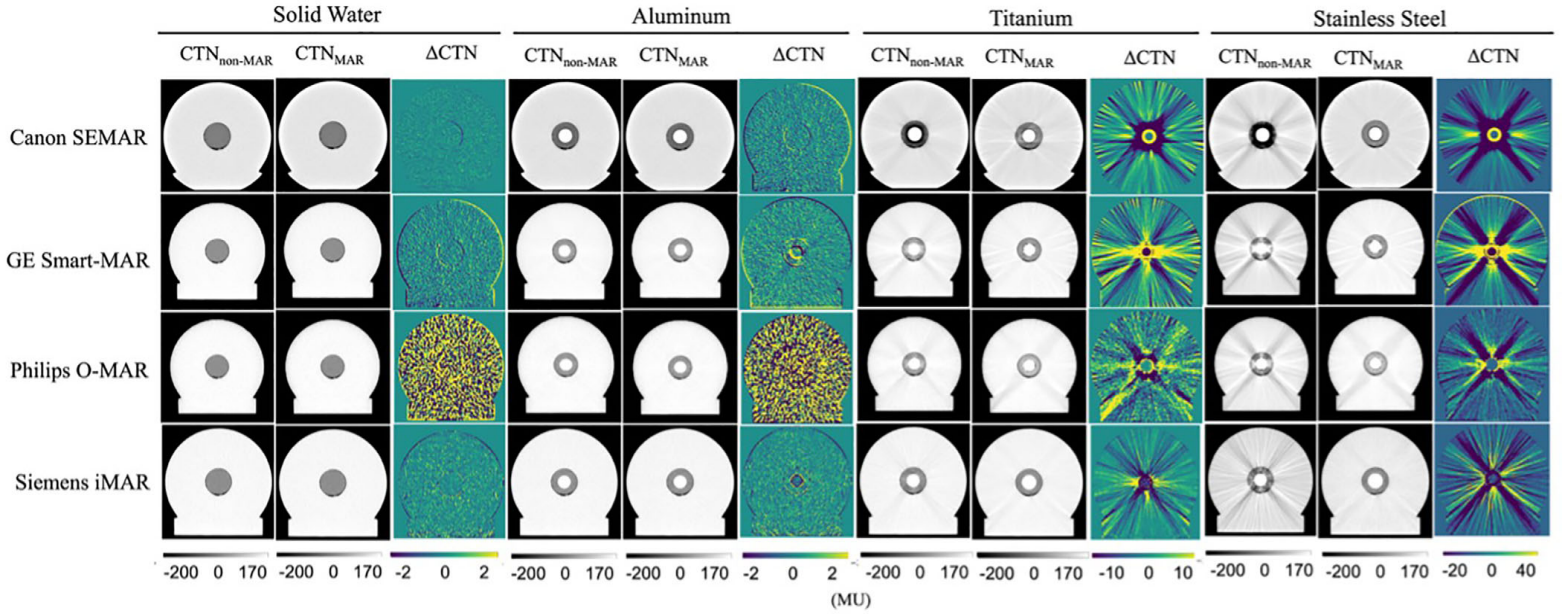
Color‐coded maps of CT axial slices showing CT numbers with and without MAR (CTNMAR and CTNnon‐MAR), alongside CT number deviations (ΔCTN) maps—calculated as CTNnon‐MAR—CTNMAR—for four insert types (solid water, aluminum, titanium, and stainless steel) across four MAR techniques (SEMAR, Smart‐MAR, O‐MAR, and iMAR). CT, computed tomography; iMAR, iterative metal artifact reduction; MAR, metal artifact reduction; O‐MAR, metal artifact reduction for orthopedic implant; SEMAR, single‐Energy metal artifact reduction.

Figure 3 presents color‐coded CTN difference maps of the phantom's cross‐section for each metal insert, visualizing artifact suppression patterns associated with four commercial MAR algorithms. These maps visualize the spatial pattern of artifact reduction introduced by each algorithm. All four MAR techniques visibly reduced the characteristic bright‐and‐dark streaks around the high‐density inserts. The effect was most apparent for stainless steel, which produced severe field‐wide streaking without MAR but showed substantially reduced distortion after correction. Overall, the maps demonstrate qualitative reduction in both artifact severity and spatial extent, providing visual support for subsequent quantitative evaluations of CTN fidelity and artifact magnitude.

### 3.2 Circular ΔCT number profile analysis

**FIGURE 4:**
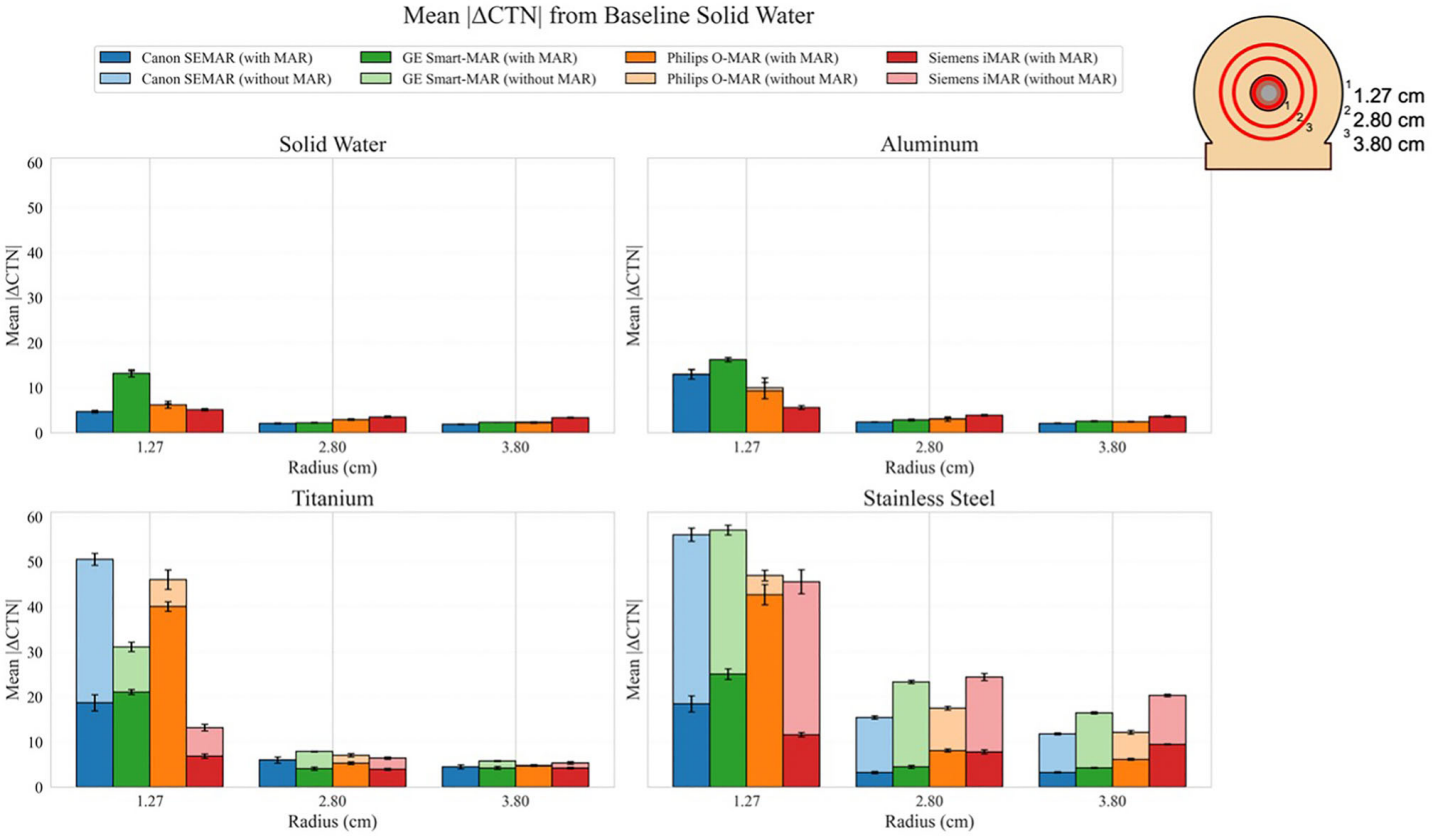
Mean absolute ΔCT number (CTN) comparison across four MAR techniques (SEMAR, Smart‐MAR, O‐MAR, and iMAR) at radial distances of 1.27 cm, 2.80 cm, and 3.80 cm from the insert center. For each technique, results are shown for both MAR‐corrected (with MAR) and uncorrected (without MAR) reconstructions. ΔCTN values represent the mean absolute difference from the corresponding baseline CTN measured in solid water. Results are grouped by material type: solid water (control), aluminum, titanium, and stainless steel. CT, computed tomography; iMAR, iterative metal artifact reduction; MAR, metal artifact reduction; O‐MAR, metal artifact reduction for orthopedic implant; SEMAR, single‐Energy metal artifact reduction.

Figure 4 illustrates the mean absolute CTN differences relative to the solid water baseline for non‐MAR and MAR‐corrected reconstructions. This metric quantifies residual artifact magnitude and indicates how closely each reconstruction approximates the expected soft‐tissue baseline. Across all materials, non‐MAR reconstructions consistently exhibited substantially higher |ΔCTN| values, especially near the insert at 1.27 cm, with largest deviations observed for higher‐attenuation materials such as titanium and stainless steel. MAR algorithms reduced these deviations to varying degrees depending on the material and algorithm. For aluminum and titanium, all four MAR techniques produced consistently low residual |ΔCTN| at mid‐ (2.80 cm) and far‐range (3.80 cm) distances, with values of approximately from 1.98 to 3.90 HU for aluminum and 3.90 to 5.27 HU for titanium. In contrast, deviations increased near the metal interface (1.27 cm), particularly for denser inserts. Smart‐MAR showed the highest |ΔCTN| for aluminum (16.83 HU), while O‐MAR yielded the largest overall |ΔCTN| for titanium and stainless steel (42.40 and 42.60 HU, respectively). O‐MAR shows a relatively small difference between the |ΔCTN| between non‐MAR and MAR, indicating limited improvement in artifact reduction.

### 3.3 Metal insert diameter accuracy

Table 2 presents segmentation‐based diameter accuracy for metal inserts. The measured post‐MAR diameters were compared with the known physical diameter of 12.7 mm to assess geometric distortion introduced by each algorithm. SEMAR and iMAR demonstrated consistently superior geometric fidelity across all materials, with percent errors remaining below 5%. O‐MAR showed material‐dependent performance: for aluminum, it exhibited 9.17% ± 0.26% error, but for titanium it achieved 0.36% ± 0.26% error–the lowest among all algorithms for this material. Smart‐MAR showed the largest deviations, with errors exceeding 19% for both titanium and stainless steel. Notably, O‐MAR was the only MAR algorithm that consistently overestimated it in both the titanium and stainless‐steel cases, although the deviation remained minimal (<3%).

**TABLE 2:** Quantitative accuracy of metal insert diameter measurements following application of four commercial metal artifact reduction (MAR) algorithms.

Metal diameter accuracy									
	Aluminum			Titanium			Stainless steel		
	Calculated diameter (mm)	Diameter error (mm)	Percentage error (%)	Calculated diameter (mm)	Diameter error (mm)	Percentage error (%)	Calculated diameter (mm)	Diameter error (mm)	Percentage error (%)
Canon SEMAR	12.35	0.35 ± 0.01	2.79 ± 0.05	12.14	0.56 ± 0.01	4.41 ± 0.08	12.27	0.43 ± 0.01	3.44 ± 0.11
GE Smart‐MAR	11.70	1.00 ± 0.05	7.85 ± 0.42	10.11	2.59 ± 0.11	20.39 ± 0.84	10.29	2.41 ± 0.08	19.03 ± 0.63
Philips O‐MAR	11.54	1.16 ± 0.04	9.17 ± 0.29	12.75	0.05 ± 0.03	0.36 ± 0.26	13.02	0.32 ± 0.04	2.55 ± 0.29
Siemens iMAR	12.25	0.45 ± 0.02	3.59 ± 0.13	12.51	0.19 ± 0.01	1.52 ± 0.10	12.30	0.40 ± 0.01	3.13 ± 0.12

Calculated diameters, absolute errors (mm), and percent errors (%) are reported for aluminum, titanium, and stainless steel inserts. Mean and standard deviation of measurements were derived from segmentation‐based measurements on five consecutive slices centered at the approximate mid‐plane of the insert for each MAR‐corrected CT scan.

### 3.4 Integrated artifact severity—*M*‐error index

We quantified artifact burden using the *M*‐error index, which combines the extent of artifact‐affected voxels with their CTN deviation from baseline. Lower *M*_error_ values indicate better artifact suppression with lower deviation from baseline. Table 3 summarizes *M*_error_ values for each MAR algorithm across the three metal inserts. In most cases, stainless steel produced the highest *M*_error_, reflecting the severity of artifacts caused by high‐density materials, followed by titanium and aluminum. SEMAR, Smart‐MAR, and iMAR yielded consistently lower *M*_error_ values, typically ranging from 0.00 to 0.83. In contrast, O‐MAR showed significantly higher *M*_error_ values, ranging from 1.15 to 2.45, indicating more extensive or intense residual artifact.

**TABLE 3:** Quantitative evaluation of residual artifact using bad pixel percentage, *M*_error_, and Δ*M*_error_ metric across the four MAR techniques (Canon SEMAR, GE Smart‐MAR, Philips O‐MAR, and Siemens iMAR).

Aluminum						Titanium				Stainless steel		
	# Of slices	Bad pixel (%)	*M*_error_	Δ*M*_error_	# Of slices	Bad pixel (%)	*M*_error_	Δ*M*_error_	# Of slices	Bad pixel (%)	*M*_error_	Δ*M*_error_
Canon SEMAR	10	0.00 ± 0.01	0.00	0.01	10	0.63 ± 0.03	0.34	0.44	10	0.58 ± 0.03	0.32	3.99
GE Smart‐MAR	24	1.23 ± 0.07	0.77	0.01	24	1.20 ± 0.07	0.83	0.26	24	1.22 ± 0.06	0.74	9.30
Philips O‐MAR	6	1.61 ± 0.10	1.15	0.05	8	2.32 ± 0.12	1.95	0.06	7	2.70 ± 0.13	2.45	5.97
Siemens iMAR	30	0.11 ± 0.01	0.05	0.01	30	0.07 ± 0.01	0.03	0.03	30	1.01 ± 0.05	0.51	12.61

Δ*M*_error_ is defined as the difference between non‐MAR and MAR *M*_error_ values.

**FIGURE 5:**
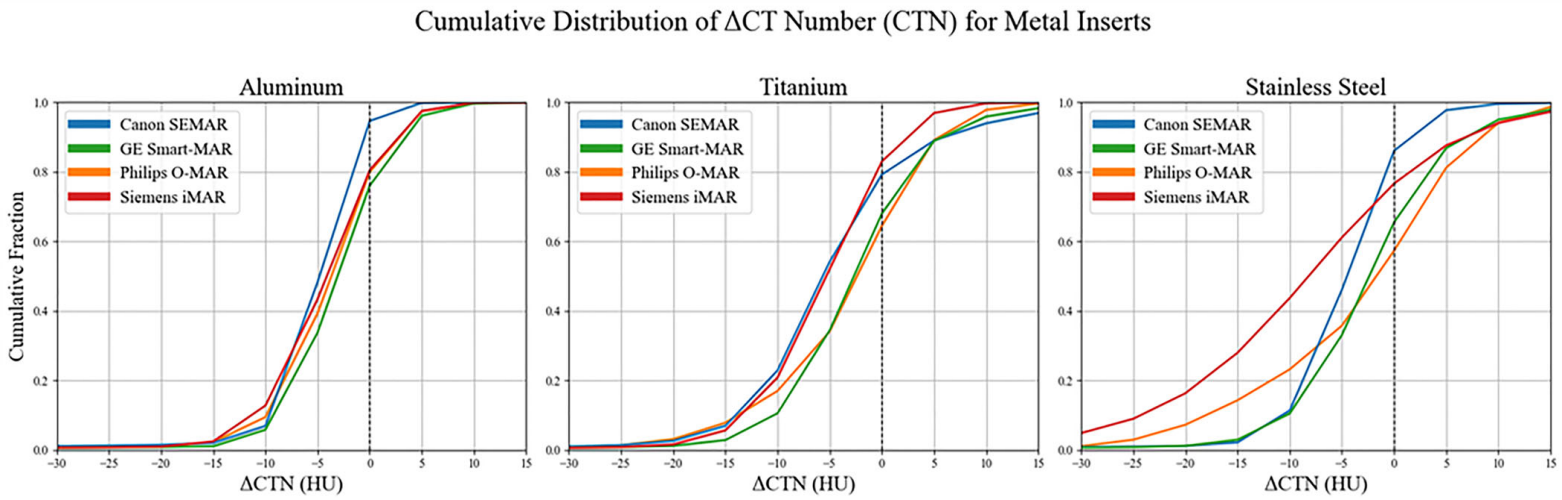
Cumulative distribution of ΔCTN values (metal—solid water) for three metal inserts—Aluminum, Titanium, and Stainless Steel—across four commercial MAR techniques: Philips O‐MAR, Canon SEMAR, GE Smart‐MAR, and Siemens iMAR. Vertical dashed lines mark ΔCTN = 0 HU as a reference for no error. CT, computed tomography; CTN, CT number; iMAR, iterative metal artifact reduction; MAR, metal artifact reduction; O‐MAR, metal artifact reduction for orthopedic implant; SEMAR, single‐Energy metal artifact reduction.

Cumulative histograms of ΔCTN within volumetric annular regions of the phantom are shown in Figure 5. Both GE Smart‐MAR and Philips O‐MAR exhibit closely clustered CTN distributions, indicating reduced artifact spread and minimal ΔCTN bias. Notably, at ΔCTN = 0, Philips O‐MAR exhibits the lowest cumulative fraction across all three metals, suggesting a greater proportion of overestimated CTN values (i.e., positive ΔCTN) relative to the other techniques. For the stainless steel insert, Canon SEMAR and GE Smart‐MAR show steeper cumulative increases in the ΔCTN range of approximately –30 to –5 HU, suggesting stronger suppression of negative artifacts compared to iMAR.

## 4 DISCUSSION

This study presents a comparative evaluation of four commercially available MAR techniques—Canon's SEMAR, GE's Smart MAR, Philips’ O‐MAR, and Siemens’ iMAR—using a custom phantom containing clinically relevant metallic inserts. Quantitative analysis across multiple metrics, including CTN accuracy, spatial distortion, and artifact spread, revealed distinct strengths and limitations among the MAR methods. Overall, SEMAR and iMAR demonstrated the most balanced performance, achieving geometric errors below 5% and consistently low *M*_error_ values across all metals. Smart‐MAR provided effective artifact suppression but exhibited diameter measurement errors exceeding 19% for titanium and stainless steel. O‐MAR showed material‐dependent performance: while achieving the highest geometric accuracy for titanium (0.36% error), it produced the largest *M*_error_ values (1.15–2.45) and highest |ΔCTN| near metal interfaces.

All four MAR techniques substantially reduced streaking artifacts and improved background uniformity relative to their respective non‐MAR reconstructions. Canon SEMAR preserved edge definition and CTN consistency in soft‐tissue‐equivalent areas, while Siemens iMAR effectively suppressed streaks with mild CTN fluctuations in low‐density regions. GE Smart MAR balanced artifact suppression with overall background correction but showed larger geometric errors for higher‐density inserts. Philips O‐MAR reduced streaking visually but showed variable performance, including overestimation of stainless‐steel insert size, likely related to mis‐segmentation or overcorrection during its segmentation‐replacement process.^14^ This size overestimation tendency has been previously documented and attributed to interpolation‐based correction strategies.^8^ These comparisons highlight the need for standardized MAR benchmarking, particularly in radiation therapy settings where CTN accuracy and geometric fidelity may affect downstream planning.

Radial profile and annular analyses showed that residual artifacts were most pronounced near metal interfaces. |ΔCTN| increased markedly at the nearest sampling distance (1.27 cm) but remained relatively low at mid and far distances, consistent with photon starvation and beam hardening effects near high‐Z materials.^1^, ^3^ Cumulative ΔCTN histograms further highlighted algorithm‐specific tendencies toward under‐ or overcorrection. These findings suggest important trade‐offs between geometric fidelity and artifact suppression, consistent with previous reports.^15^, ^16^, ^17^ SEMAR and iMAR maintained more consistent diameter accuracy and edge definition, whereas O‐MAR showed stronger evidence of near‐metal blurring despite high titanium diameter accuracy (0.36% error), suggesting potential over‐smoothing in the segmentation‐replacement process. The optimal algorithm choice may therefore depend on the specific clinical task—whether target volume delineation requiring sharp edges or background uniformity requiring aggressive artifact suppression is prioritized.

The observed differences may be relevant to radiation therapy workflows, although this study does not directly evaluate dose calculation or contouring outcomes. Near metal interfaces, O‐MAR produced |ΔCTN| of 42.40–42.60 HU, exceeding a threshold associated with clinically significant dose calculation errors.^3^ This suggests that patients with orthopedic hardware or other high‐Z implants may require further evaluation in treatment planning workflows. In contrast, the more consistent performance of SEMAR and iMAR across metrics suggests potential advantages when both geometric accuracy and artifact suppression are important.

Several limitations should be acknowledged. First, this study evaluates the integrated performance of each vendor's MAR implementation as clinically deployed; therefore, observed differences reflect both algorithm behavior and scanner‐specific acquisition parameters. Scanner‐specific parameters including slice thickness (1.00–3.00 mm), reconstruction kernel, and field of view varied across systems and may have contributed to inter‐vendor differences. Second, inferential statistical comparisons such as *t*‐test were not performed due to limited sample size; results are presented descriptively for benchmarking purposes. Third, although phantom studies cannot fully reproduce anatomical complexity of clinical scenarios, they provide controlled comparisons that are otherwise impossible to obtain due to patient‐to‐patient variability. Finally, this study does not include treatment planning system (TPS)‐based dose calculations or contouring evaluations. Therefore, the presented HU‐based metrics should be interpreted as indirect indicators of potential downstream impact rather than direct measures of clinical dose accuracy.

The single‐insert phantom design was intentionally selected to isolate algorithm‐specific behavior and avoid confounding from overlapping artifacts or multi‐object scatter. This approach improves reproducibility and enables direct comparison across vendors, but it also limits clinical realism. MAR performance may differ for larger or more complex implants, such as bilateral hip prostheses, spinal instrumentation, or dense dental restorations. In addition, algorithms may be optimized for different clinical scenarios; for example, O‐MAR was originally developed and validated for large orthopedic implants, where artifact characteristics differ from the single‐insert geometry used here. Therefore, relative performance rankings should be interpreted cautiously.

Future work should extend this benchmarking framework to radiation therapy‐specific endpoints, including TPS‐based dose calculation accuracy and contouring variability near metal implants. Additional studies could also evaluate patient datasets or anthropomorphic phantoms and compare performance against established artifact metrics such as the robust metal artifact index proposed by Cammin et al.^18^ As metal implants become increasingly prevalent among radiation therapy patients, the systematic benchmarking of MAR techniques remains critical for improving both image quality and dosimetric accuracy across clinical contexts.

## 5 CONCLUSION

This study provides a controlled comparison of four commercial MAR algorithms—SEMAR, Smart‐MAR, O‐MAR, and iMAR—using standardized quantitative metrics relevant to radiation oncology. SEMAR and iMAR achieved the most consistent geometric accuracy (<5% error) and artifact suppression across all metals, while O‐MAR demonstrated material‐dependent performance with substantial residual artifacts near metal interfaces. This study provides a vendor‐neutral, quantitative benchmarking framework for evaluating MAR algorithm performance under controlled conditions. The findings offer system‐level insights into algorithm behavior and should be interpreted as a basis for hypothesis generation, with further validation required in clinically realistic scenarios.

## AUTHOR CONTRIBUTIONS

**Beechui Koo**: Methodology; data analysis; manuscript writing. **Daehong Kim**: Data analysis. **Hyunuk Jung**: Data acquisition. **Mitchell Polizzi**: Experiments. **Indra J. Das**: Manuscript review. **Siyong Kim**: Supervision. **James J. Sohn**: Study design and supervision.

## ETHICS STATEMENT

This study did not involve human participants or animal subjects; therefore, ethical approval was not required.

## CONFLICT OF INTEREST STATEMENT

The authors declare no conflicts of interest.

## Data Availability

The data that support the findings of this study are available from the corresponding author upon reasonable request.

## References

[1] Boas FE , Fleischmann D . CT artifacts: causes and reduction techniques. Radiographics. 2012;32(2):767‐787.22582358

[2] Katsura M , Sato J , Akahane M , Kunimatsu A , Abe O . Current and novel techniques for metal artifact reduction at CT: practical guide for radiologists. Radiographics. 2018;38(2):450‐461. doi:10.1148/rg.2018170102 29528826 10.1148/rg.2018170102

[3] Huang JY , Followill DS , Howell RM , et al. Approaches to reducing photon dose calculation errors near metal implants. Med Phys. 2016;43(9):5117‐5130. doi:10.1118/1.4960632 27587042 10.1118/1.4960632 PMC4991994

[4] Tanoue S , Kidoh M , Oda S , et al. Impact of single‐energy metal artifact reduction on CT image quality in patients with dental hardware. Comput Biol Med. 2018;103:101‐108. 30384174 10.1016/j.compbiomed.2018.10.023

[5] Feldhaus F , Böning G , Jonczyk M , et al. Metallic dental artifact reduction in computed tomography (Smart MAR): improvement of image quality and diagnostic confidence in patients with suspected head and neck pathology and oral implants. Eur J Radiol. 2019;118:153‐160. doi:10.1016/j.ejrad.2019.07.015 31439235 10.1016/j.ejrad.2019.07.015

[6] Weiss J , Schabel C , Bongers M , et al. Impact of iterative metal artifact reduction on diagnostic image quality in patients with dental hardware. Acta Radiol. 2017;58(3):279‐285. doi:10.1177/0284185116646144 27166346 10.1177/0284185116646144

[7] Hilgers G , Nuver T , Minken AW . The CT number accuracy of a novel commercial metal artifact reduction algorithm for large orthopedic implants. J Appl Clin Med Phys. 2014;15(1):4597. doi:10.1120/jacmp.v15i1.4597 24423859 10.1120/jacmp.v15i1.4597 PMC5711242

[8] Gjesteby L , De Man B , Jin Y , et al. Metal artifact reduction in CT: where are we after four decades?. IEEE Access. 2016;4:5826‐5849. doi:10.1109/ACCESS.2016.2608621

[9] Chou RH , Chi HY , Lin YH , Ying LK , Chao YJ , Lin CH . Comparison of quantitative measurements of four manufacturers’ metal artifact reduction techniques for CT imaging with a self‐made acrylic phantom. Technol Health Care. 2020;28(S1):273‐287. doi:10.3233/THC‐209028 32364160 10.3233/THC-209028 PMC7369061

[10] Huang JY , Kerns JR , Nute JL , et al. An evaluation of three commercially available metal artifact reduction methods for CT imaging. Phys Med Biol. 2015;60(3):1047‐1067. doi:10.1088/0031‐9155/60/3/1047 25585685 10.1088/0031-9155/60/3/1047 PMC4311882

[11] Kalender WA , Hebel R , Ebersberger J . Reduction of CT artifacts caused by metallic implants. Radiology. 1987;164(2):576‐577. doi:10.1148/radiology.164.2.3602406 3602406 10.1148/radiology.164.2.3602406

[12] Wang Y , Zhang Y , Ma J , et al. Metal artifact reduction in CT with deep learning technique: current status and future prospects. J Appl Clin Med Phys. 2021;22(6):11‐27. doi:10.1002/acm2.13121

[13] Sohn JJ , Polizzi M , Richeson D , Gholami S , Das IJ , Song WY . A novel workflow with a customizable 3D printed vaginal template and a direction modulated brachytherapy (DMBT) tandem applicator for adaptive interstitial brachytherapy of the cervix. J Clin Med. 2022;11(23):6989. doi:10.3390/jcm11236989 36498563 10.3390/jcm11236989 PMC9738087

[14] Jeong S , Kim SH , Hwang EJ , Shin CI , Han JK , Choi BI . Usefulness of a metal artifact reduction algorithm for orthopedic implants in abdominal CT: phantom and clinical study results. AJR Am J Roentgenol. 2015;204(2):W146‐W153. doi:10.2214/AJR.14.12745 10.2214/AJR.14.12745 25615752

[15] Subhas N , Primak AN , Obuchowski NA , et al. Iterative metal artifact reduction: evaluation and optimization of technique. Skeletal Radiol. 2014;43(12):1729‐1735. doi:10.1007/s00256‐014‐1987‐2 25172218 10.1007/s00256-014-1987-2

[16] Zhang Y , Pu YF , Hu J , Liu Y , Zhou JL . A new CT metal artifacts reduction algorithm based on fractional‐order sinogram inpainting. J Xray Sci Technol. 2011;19(3):373‐384. 21876286 10.3233/XST-2011-0300

[17] Meyer E , Raupach R , Lell M , Schmidt B , Kachelrieß M . Normalized metal artifact reduction (NMAR) in computed tomography. Med Phys. 2010;37(10):5482‐5493. doi:10.1118/1.3484090 21089784 10.1118/1.3484090

[18] Cammin J . A robust index for metal artifact quantification in computed tomography. J Appl Clin Med Phys. 2024;24(6):e14453. doi:10.1002/acm2.14453 10.1002/acm2.14453 PMC11302826 38923797

